# Somewhere to go: a position paper on addressing gaps in transition care for adults with childhood-onset rare diseases

**DOI:** 10.1186/s13023-025-03973-0

**Published:** 2025-10-02

**Authors:** Laura L. Tosi, Tracy S. Hart, Gabriela Beug, Erika Carter, Eleanor M. Perfetto

**Affiliations:** 1 Division of Orthopaedic Surgery & Sports Medicine, Children’s National Hospital, 111 Michigan Ave NW, Washington, 20010 DC USA; 2 Osteogenesis Imperfecta Foundation, Gaithersburg, MD USA; 3 Professor Emerita, School of Pharmacy, University of Maryland, College Park, MD USA

**Keywords:** Transition of care, Adult rare disease care, Childhood-onset rare disease

## Abstract

Adults with childhood-onset rare disease face many challenges when transitioning from pediatric services to adult care. While they often received specialized pediatric care, the adult healthcare system provides few resources for those whose rare disease began in childhood. Many adult care providers are hesitant to take on adults with rare disease and may shunt them elsewhere. Treatment recommendations are evolving rapidly, making it difficult for any clinician to stay up to date, and patients with rare disease often have special needs that must be addressed by multiple specialists at once. These young adults are often faced with “nowhere to go” for care.

A pressing question is: What can be done now to help adult patients with rare diseases gain access to the care they need. In response, a patient-community-driven, solution-oriented conference was held, entitled, “Somewhere to Go for Adults with Childhood-Onset Rare Diseases: A Conversation About How We Can Fill Gaps in Care.” The purpose was to bring the rare-disease community together to explore action steps that could be taken in the next two to three years to address the growing, national care crisis for adults with rare diseases transitioning from pediatric to adult care. This position paper provides an overview of the key topics discussed and summarizes the 21 prioritized, actionable recommendations produced.

**Supplementary Information:**

The online version contains supplementary material available at 10.1186/s13023-025-03973-0.

## Background

An estimated 30 million Americans live with a rare disease, defined by the 1983 Orphan Drug Act as one person diagnosed among 200,000 people or fewer in the US [1, 2]. For the majority of patients, their rare disease began in childhood [3]. Although they may have received specialized pediatric care, the adult healthcare system provides few resources for most adults whose rare diseases began in childhood [4]. Thus, millions of young adult and adult patients face difficulties finding knowledgeable primary care and suitable specialty support [5, 6].

The number of identified rare diseases is rapidly increasing [7]. In 2016, the NIH identified 7,000 rare diseases; recent estimates place that number closer to 10,000 [8, 9]. More than 95% of thes e disorders have no Food and Drug Administration (FDA) -approved treatment [10]. However, with improved care strategies, and in some cases, novel therapies, many who previously would have had a short life expectancy, now live into adulthood [4, 11]. But, the healthcare system has not evolved and adapted accordingly [12–14].

Several key elements make healthcare access problematic for adults with childhood-onset rare diseases, who represent approximately 70% of the rare-disease community [3]. Both adult primary-care providers and specialists often lack training and experience with these diseases [15, 16]. They face time and financial constraints providing care and may encounter administrative burdens in doing so, which makes them hesitant to take on adults with rare disease [17]. In addition, adult providers may have minimal communication with patients’ pediatricians [18]. Thus, they have limited access to a patient’s medical history, impeding continuity of care and decision-making [16]. In addition, buildings in which they practice may be inaccessible to some patients. Many adult providers attempt to shunt them elsewhere [19]. Exacerbating the challenges, recommendations for treatment are evolving rapidly as scientific understanding advances, making it difficult for any clinician to stay up to date. Moreover, patients with rare disease often have special needs that must be addressed by multiple specialists at once [20]. While multidisciplinary care clinics are a hallmark of pediatric care, these clinics are rarely seen in adult-care facilities [16].

Patients with rare diseases are not alone when facing barriers to access and quality care as they transition to adulthood. The National Alliance to Advance Adolescent Health, which developed the Got Transition^®^ [21] program, recently partnered with the George Washington University Fitzhugh Mullan Institute for Health Workforce Equity to underscore similar challenges faced by young adults with more common -- but also complex -- medical conditions [22]. Its recent White Paper, “Strengthening the Adult Primary Care Workforce to Support Young Adults with Medical Complexity Transitioning to Adult Health Care,” underscores the need for change in workforce training, payment/reimbursement for care, research, and coalition building for patients with complex disorders [23] (see Appendix 1). There is significant overlap in the needs of the rare-disease and complex-medical-conditions communities.

Cystic fibrosis (CF) and sickle-cell disease (SCD), offer established transition-of-care models that may help address the broader challenges faced by the rare-disease community. The Cystic Fibrosis Foundation promotes gradual, deliberate transfer of responsibility from caregivers to patients, supporting clear communications among pediatric and adult care teams, patients, and families, to enhance quality of life, promote patient independence, and ensure continuity of care during the transition from pediatric to adult care [24, 25]. The established sickle cell disease (SCD) transition programs also help adolescents and young adults shift from pediatric to adult healthcare [26–31]. 

These models demonstrate that integrating disease-specific guidelines, multidisciplinary coordination, early transition planning, and patient empowerment can mitigate transition barriers. Resource constraints may limit application of such models to many rare diseases, but the principles of collaborative care, proactive planning, and patient-centered tools, are adaptable and offer a framework for developing scalable transition strategies across the rare-disease community.

Progress on these issues will take time. A pressing question is: “What can be done now?” In response, the Osteogenesis Imperfecta Foundation (OIF) organized a patient-community-driven, solution-oriented conference entitled, “Somewhere to Go for Adults with Childhood-Onset Rare Diseases: A Conversation About How We Can Fill Gaps in Care,” in October 19–20, 2023. The purpose was to bring the rare disease community together to explore action steps that could be taken in the next two to three years to address the growing national care crisis with regard to pediatric-to-adult care transition for people with rare diseases. The desired output was a list of prioritized actions the group could take on as a whole or in smaller groups as future spinoff activities. This position paper provides an overview of the key discussions and summarizes the 21 actionable recommendations produced with three key priorities .

## Methods

The conference was organized by the OI Foundation and held as a hybrid (in-person and online) model to encourage increased participation and breadth of dialogue. It examined current rare-disease care infrastructure; the role of technology in providing quality care; payment-model issues; patient-engagement issues, and current care models including centers of excellence and existing consortium and network models. Attendees included patients, caregivers, health professionals, patient-advocacy leaders, government, and industry representatives, and OIF staff.

The meeting sought to: (1) Bring together a multistakeholder, multidisciplinary group for an interactive, working meeting to explore the challenges faced by adult patients with rare, childhood-onset diseases who find themselves with “nowhere to go” for care; (2) Enumerate and describe the current gaps, barriers, and challenges these patients face and the potential causes behind them; (3) Think strategically about solutions to address these gaps, barriers, and challenges; and (4) Prioritize which issues and/or solutions could be addressed collaboratively over the next 2–3 years by members of the assembled group and other potential partners.

The agenda was organized around four main session topics (See full Agenda in Appendix 2) described below). Each began with speakers briefly introducing the care transition topic by providing firsthand experiences, describing challenges and barriers, discussing possible solutions, and offering examples of practices and models that might be scaled, replicated, and/or disseminated. To promote group interaction, stakeholder engagement, and co-creation of deliverables, a facilitated, open discussion among all participants followed speaker remarks for each session, during which participants were asked to answer the following questions:

- What are the top 3 priorities that must be addressed in the next 2–3 years?
- What are 3 specific things we can do *collectively now* to achieve this vision?
- What does a coalition look like that will work *collaboratively now* to design and move a plan forward in the next 2–3 years?

In discussing these questions, participants were asked to consider how we can best meet the needs of underserved populations and the role patient organizations should play.

All group discussions were audio recorded and summarized in real-time in a series of flipcharts. Participants were able to comment and provide edits during the discussions. At the end of Day 1, discussion points from all sessions were consolidated into a strawman list for group discussion on Day 2. The consolidated list was refined during Day 2 group discussions to ensure clarity, refine language, and lump and split the list of proposed actions. The final list was prioritized using sticker-dot voting, with each individual having three equally weighted votes. The group was asked to review the prioritized list and suggest any changes needed before the list of recommendations was finalized.

### Key issues

#### Envisioning the 21st century: centers of excellence (COEs) for persons with rare Disease – What does that look like and how do we get there??

The critical need for structured transitions from pediatric to adult care is a major concern, as many young adults with rare diseases face a “bridge to nowhere” when aging out of pediatric systems. A successful COE model would not only provide specialized medical care but also integrate caregiver support, academic and career counseling, financial and legal assistance, transportation services, and mental health resources. While COEs offer significant potential, challenges such as financial sustainability, standardization, and the lack of trained adult care providers remain.

Experts debated whether the future of COEs should be brick-and-mortar institutions or hybrid models leveraging telemedicine, provider networks, and mentorship programs to expand expertise and improve access to care. Certification models for specialized centers were presented as a means to ensure consistency in care delivery, workforce training, and access to critical services, such as cardiac care, clinical trials, and assistive technology. However, systemic barriers persist, with adult providers often unprepared to manage adults with childhood-onset rare diseases and reimbursement models failing to reflect the complexity of care required. The experts underscored the need for interdisciplinary collaboration, proactive transition planning, and dynamic, patient-centered care models that adapt to evolving needs, ensuring that individuals with rare diseases continue to receive comprehensive support throughout their lives.

### Evidence for Decision-Making: leveraging patient data and patient experience

Evidence is needed to support decision-making with regard to transitions from pediatric to adult care. However, there are data challenges. Natural history studies, clinical trials, and a focus on patient-reported outcomes are critical for ensuring that treatments are both effective and aligned with patient needs. The importance of longitudinal data collection was emphasized, highlighting how this can help researchers understand disease progression, improve trial design, and create meaningful clinical endpoints. The experts also explored patient-focused medical product development, a framework ensuring that patient perspectives, needs, and priorities are integrated throughout the development and evaluation of medical treatments.

Challenges include ensuring the accuracy and completeness of electronic health records, reducing data variability in clinical trials, and optimizing the role of patient engagement in improving research outcomes remain a concern. Other concerns were raised about regulatory barriers and outdated gold standards in trial design, particularly the limitations of randomized controlled trials for rare diseases.

A key theme was data collaboration—the need for institutions, researchers, and advocacy groups to harmonize disparate data sources to improve the quality and relevance of research. Emphasis was on the need for interdisciplinary collaboration, early patient involvement in research design, and policy changes to support data-driven, patient-centered decision-making in rare disease research and treatment.

### Innovation in care coordination for patients with rare diseases and complex needs

There are numerous challenges when navigating fragmented healthcare systems, transitioning from pediatric to adult care, and accessing specialists across dispersed networks. Telehealth and virtual nursing care can be key solutions to bridge gaps in care, particularly for those in rural areas or requiring specialized expertise. The importance of integrating Medicaid managed care with long-term support services can ensure continuity and accessibility for individuals with intellectual and developmental disabilities (IDDs). A peer-to-peer disease management model can be an effective way to enhance patient adherence, reduce hospitalizations, and provide long-term support at no cost to patients. The experts underscored the need for interdisciplinary collaboration, policy changes, and scalable innovations to streamline care coordination and improve outcomes for rare disease communities.

## Coalitions, collaboratives, and working together

Progress in improving care transitions for patients with childhood-onset rare diseases has been very limited. The power of collaboration is important to advancing policy changes, improving care transitions, and strengthening the rare disease workforce. Coalitions create opportunities for advocacy, drive systemic change, and amplify the patient voice in policy development. The importance of empowering young adults as immediate stakeholders in public policy was emphasized, recognizing their lived experiences as essential in shaping the future of rare disease advocacy.

Speakers discussed how care transitions go beyond medical needs, requiring a holistic approach that includes employment, education, and long-term social support. The need for strong partnerships uniting advocacy groups, government agencies, and healthcare institutions was underscored, as these collaborations enhance resource sharing, influence policy, and improve access to care. The session concluded with an overview of the National Partnership for Pediatric to Adult Care Transition (NPPACT) [32], a coalition dedicated to addressing systemic barriers in workforce development, healthcare funding, and policy solutions for care transitions. This session reinforced the idea that working together across sectors is crucial to achieving meaningful, lasting change for the rare disease community.

The conference discussion and prioritization exercise produced a list of 21 prioritized issues with actionable steps (See Table 1). The list includes addressing critical gaps in care for adults with childhood-onset rare diseases, leveraging multidisciplinary collaboration, improving care coordination, and increasing advocacy efforts to ensure progress over the next 2–3 years.

**Table 1:** Top priority action steps to be address in 2024–2026 to smooth care transitions

Topic	
1.	**Understand the Current Landscape of Transition Care.** Gain better understanding of all that is happening in transition care. Develop an inventory/catalogue (who is doing what and where, that can be joined, built on, leveraged, disseminated, scaled).
2.	**Develop transition success measures**. If these measures are to be truly impactful, they must go beyond structure and process measures and also include measures of health improvement and patient satisfaction.
3.	**Better leverage the use of telemedicine to improve transitions.** Expanding the use of telemedicine can further enhance accessibility of care, allowing patients to maintain continuity of care through virtual consultations and remote monitoring, particularly in underserved regions.
4.	**Build and better leverage the workforce needed for the future to support transition care**. Systemic changes are necessesary to ensure that primary care providers are better equipped to manage the needs of transitioning patients. This includes enhancing medical training for adult primary-care providers, integrating transition care into routine practice to ensure continuity and accessibility, and incentivizing healthcare providers to specialize in transition care. Policy, advocacy and funding initiatives must be advanced to sustain this workforce.
5.	**Develop an accessible toolbox.** Improve patient (and family/caregiver) preparedness/readiness. to help provide a soft landing for transitions.
6.	**Advance transitions as a policy issue; develop a policy agenda.** Transition care must be integrated into federal and state healthcare policies. Advocacy efforts should focus on securing funding for transition programs, incentivizing providers to specialize in transition care, and addressing insurance barriers (reimbursement challenges, Medicaid/Medicare coordination).
7.	**Develop standards and advance integration of EHRs with other sources to strengthen evidence generation and dissemination of information**. Lack of interoperability among EHR systems leads to fragmented medical records, making it difficult for providers to access a patient’s full health history, especially for those with rare diseases who receive care from multiple specialists. To address these challenges, healthcare institutions must establish national data-sharing standards, improve interoperability between systems, and ensure patients can access and verify their own medical records.
8.	**Develop a research agenda on transitions**. A structured research agenda is important to support evidence-based decision-making in transition care. Key areas include evaluating care models, measuring patient outcomes, and conducting economic impact assessments. Longitudinal studies and quality improvement initiatives can assess transition success by collecting baseline data before interventions and tracking key health and social outcomes.
9.	**Address reimbursement**,** insurance**,** coding issues**. Gaps in insurance coverage, low reimbursement rates, and complex billing codes lead to gaps in care, delays in treatment, and financial burdens on patients and families. Medicaid and Medicare coverage limitations often lead to care disruptions and financial strain, and coding issues make it difficult for providers to bill for transition-related services. Policy reforms must focus on increasing reimbursement rates, simplifying coding structures, and ensuring coverage continuity for transitioning patients. Expanding value-based payment models and incentivizing providers to specialize in transition care can improve access and outcomes.
10.	**Improve the lack of standards**,** guidance**,** clinical practice guidelines**,** quality of care measurement.** There is a need for clear clinical practice guidelines to establish best practices for transitioning patients.
11.	**Improve mental health support during transitions.** Young adults moving to adult care may struggle with the emotional burden of leaving familiar pediatric providers, navigating new healthcare systems, and handling increased self-management responsibilities among other responsibilities​. Solutions include integrating mental health screenings and support services into transition programs, ensuring access to counseling, peer support, and community-based mental health resources.Expanding provider training on transition-related mental health challenges and creating dedicated mental health transition coordinators were identified as key strategies to improve long-term patient well-being.
12.	**Improve lack of care coordination throughout and after transitioning**. Lack of structured care coordination in transition care leads to fragmented services, miscommunication among providers, and increased burden on patients and families. Virtual nursing and telehealth models are potential solutions. Additionally, care management models that integrate medical, social, and long-term support services can streamline communication between providers and help patients transition more smoothly into adult care.
13.	**Leverage AI and other technologies to support transitions.** AI can be used to develop and improve telehealth, virtual nursing, and electronic health record integration.
14.	**Address transition fatigue (from so many kinds of transitions at once).** Many patients experience multiple transitions simultaneously, which can be overwhelming. Recommendations include phased transitions and better communication between healthcare providers.
15.	**Better support caregivers/family members/friends/care team/advocates throughout transitions.** Training, counseling, and support resources must be provided to the support team of the individual to avoid caregiver burnout and emotional burden.
16.	**Support for better-inform decisions about which provider(s) are needed when.** There is often lack of clarity about who drives decisions and when to use specialists. This relates to lack of evidence, guidelines, and care models, as well as efficiently address workforce issues.
17	**Pilot**,** expand on novel care models.** Successful care models from different rare disease communities should be evaluated, scaled, and adapted for broader implementation.
18.	**Increase and improve appropriate diagnosis**. This is needed to ensire prevalence, evidence, cost estimates are sound. Might we be looking at the tip of the iceberg?
19	**Consider the impact on transitions of culture-of-care differences between adult and pediatric care.** Pediatric care is typically family-centered, highly coordinated, and focused on holistic support, while adult care places more responsibility on the patient for self-management and navigating the healthcare system independently.
20.	**Consider stigma as part of transitions.** Stigma associated with disability and rare diseases often impacts access to quality care. There is a need for awareness campaigns and advocacy efforts to address this issue.
21.	**Consider the many different transitions happening over time: pediatric to adult to geriatric; Medicare to Medicaid.** Healthcare transitions extend beyond pediatric-to-adult care, continuing into geriatric care and insurance shifts like Medicaid to Medicare, often leading to care disruptions and provider gaps. Patients with childhood-onset conditions must repeatedly navigate new healthcare systems, facing eligibility changes and coverage limitations that impact access to specialized care. Addressing these challenges requires better care coordination, policy reforms, and integrated transition planning to ensure continuous, best care practices throughout life.

## Discussion

Conference attendees emphasized challenges in care transitions, including the lack of appropriate adult care models, coordination challenges, provider knowledge gaps, and systemic policy barriers. They concurred that building coalitions among patients, caregivers, advocacy groups, clinicians, policymakers, and researchers will be necessary to improve care transitions for patients with childhood-onset rare diseases. It will be essential for coalitions to work with policymakers to expand and revise training programs, enforce ADA compliance, improve insurance options, and advocate for systemic changes in care models.

### Enhancing patient empowerment

The conference was patient-organization led, and numerous patients and caregivers were among the invited participants, fully contributing their perspectives into the 21 issues. The group discussed that none of the 21 issues can be addressed by any one stakeholder group or organization, that collaboration among stakeholders will be required, and that patient and caregiver involvement is assumed in all efforts to address the 21 issues.

While patient empowerment (or lack thereof) was not specifically listed as one of the issues, it was a key theme throughout all discussions and is a common thread among the issues, directly and indirectly. For example, “Number 5, develop an accessible toolbox to improve patient (and family/caregiver) preparedness/readiness,” and “Number 11, improve mental health support during transitions,” can directly enhance patient empowerment. Whereas, “Number 8, develop a research agenda on transitions,” is more indirect. Patients, caregivers, and families should be engaged in any efforts to develop a transitions research agenda to ensure the research meets their needs and addresses outcomes of importance to the patient community. This also enhances patient empowerment.

#### The healthcare system is failing adults with childhood-onset rare diseases

The current healthcare system is designed to care for adults who become disabled later in life, typically from common diseases of aging, not those who grow up with complex, rare diseases or disabilities. Conference participants supported and fully concurred with the GotTransition^®^ document that emphasizes long-term goals for workforce education and training, payment policies, research, and coalition building to support patients with complex medical conditions entering the adult healthcare system. The group acknowledged that many of these goals will take years to accomplish. While there is significant overlap between patients with complex disorders and those with rare diseases, the complexity and uniqueness of rare disorders require an expanded set of priorities. Participants focused on identifying goals and activities that could motivate change and improve access and quality over the short term.

#### Pediatric care models for patients with rare diseases cannot provide lifelong support, and adult healthcare was not designed for individuals with childhood onset rare diseases

Effective adult-care models and facilities for adults with childhood-onset rare diseases are sparce, with diseases like cystic fibrosis and sickle cell disease as exceptions. Care is generally provided in silos. Redesigning care to ensure continuity as patients age will require innovation to develop interdisciplinary, patient-centered care models. While centers of excellence might be preferred, they may prove financially unsustainable and geographically inaccessible for many patients. Telemedicine can help overcome distance obstacles, but given the complexity of rare diseases, in-person care will often be required. Moreover, recent research suggests telemedicine is primarily used by individuals who already have access to healthcare [33, 34]. Standardized protocols, guidelines, and cadence-of-care documents have extended patient lifespans, notably in muscular dystrophy and cystic fibrosis [35, 36]. However, some disorders are so rare that the data needed to create such documents has not yet been assembled. Insurance barriers, the lack of specialized adult providers, and gaps in legal support for patients with intellectual disabilities further complicate the difficulties in transitioning patients with rare disease into adult healthcare.

#### To better understand the needs of rare disease patients, we must solicit their views of what’s most important and assemble data that allow us to explore those insights

Improved data sharing and a focus on patient-centered outcomes will help inform healthcare decisions and development of new care models. However, existing data need to be examined with a critical eye, as early natural history studies primarily included only the perspective of clinicians. Moreover, natural history studies can be expensive and out of reach for small organizations. Yet, natural history studies are beginning to play a role in clinical-trial design, as disease-progression data provide a standard against which drug success can be measured. The National Organization for Rare Diseases (NORD) is actively enhancing data sharing and patient-reported outcomes in the rare disease community. Through its IAMRARE Registry Program [37] NORD collaborates with patient and research communities to collect patient-reported data, thereby advancing real-world evidence and supporting the development of new therapies. Additionally, NORD has partnered with the Critical Path Institute and the FDA to establish the Rare Disease Cures Accelerator-Data and Analytics Platform [38], an FDA-funded initiative to develop centralized infrastructure designed to host and share de-identified data.

There is a need for interoperable electronic health records (EHRs). Disconnects in patient data hinders research and coordinated care. There is hope that AI will improve EHRs and reduce the burden of collecting data for natural history studies. In addition, collaborative data-sharing initiatives can create a continuous learning system that integrates patient input into medical innovation.

### Dealing with the present

With over 10,000 identified rare diseases, it is not feasible for medical providers to have in-depth knowledge of each [39]. This underscores the need for easily accessible resources and tools that aid in care delivery. Patients and their families need to stay up-to-date on research advancements, playing an active role in managing patients’ health and helping create an improved delivery system. Patient organizations, such as the OIF, offer invaluable educational resources designed to empower patients. By utilizing these resources, patients can better understand their condition and advocate for their needs, ultimately fostering more productive and informed interactions with their healthcare teams.

#### There are several key actions the rare-disease community can initiate over the short term

The 21 actionable steps are ambitious and comprehensive. While all 21 are critical in some way, it is not feasible for the rare-disease community to tackle all 21 simultaneously. Participants concurred that a strategic, phased approach will be needed for implementation. To build momentum and create immediate impact, attendees agreed to focus on the three highest-priority actions: understanding the current landscape of transition care, developing transition success measures, and expanding telemedicine. The attendees expressed that, over the next few years, partnerships, coalitions, and collaborations should address the following:

#### Understanding the current landscape of transition care

The advocacy community needs to take a leadership role in collating all that is happening in pediatric-to-adult care transitions for individuals with rare diseases. It will be essential to develop an inventory of who is doing what and where, so that successful efforts can be disseminated, built on, joined, and scaled. While the initial focus should be on transition of care from pediatric to adult care, over the longer term, lifespan issues must be addressed. Also, understanding what is out there, available, and working for patients can help create a pipeline of practical, successful resources for creating a transition toolbox of resources made available to patients, families and clinicians.

#### Development of transition success measures

Participants concluded that developing measures of success that can help define what is working and what is not could serve as an impetus for change. If these measures are to be truly impactful, they must go beyond structure and process to also include outcome measures of health improvement and patient satisfaction. Developing these measures will also help create a research agenda outlining the work that still needs to be done to establish care standards.

#### Expanding telemedicine

Expanding the use of telemedicine can further enhance accessibility of care, allowing patients to maintain continuity of care through virtual consultations and remote monitoring, particularly in underserved regions. Technology and telemedicine can afford access for adult patients that they do not have now to specialists with rare disease expertise. Telemedicine offers financial benefits such as minimizing travel expenses and burden on patients and families.

### Limitations

The most important limitation of this work is that we relied on the input of a convenience sample of invited meeting attendees. However, the attendees represented a broad range of stakeholders, backgrounds, rare diseases, disciplines, and patients and families. In addition, our meeting only focused on adults with childhood-onset rare diseases. There are overlapping care transition problems faced by children with complex disorders who are moving into adulthood as well as adults who develop rare diseases after moving into adulthood. These were not specifically addressed.

## Conclusion

The meeting participants identified 21 issues that represent gaps, barriers, and challenges when it comes to pediatric-to-adult care transitions and suggested solutions to address them. While it may take many years to address all 21, there are real opportunities for patient-advocacy organizations to have real impact in the short term by focusing on the conference’s three highest-priority recommendations: (1) Understanding the current landscape of transition care, (2) Development of transition success measures, and (3) Expanding telemedicine. It will take strong coalitions of patients with rare diseases, their caregivers, clinicians, advocacy organizations, payers, and policymakers to address the issues and make the solutions real to smooth pediatric-to-adult care transitions for the rare disease community.

## Electronic supplementary material

Below is the link to the electronic supplementary material.

Supplementary Material 1

## Data Availability

Data sharing is not applicable to this article as no datasets were generated or analysed during the current study.

## Abbreviations

ADA: Americans with Disabilities Act
AI: Artificial Intelligence
CMS: Centers for Medicare & Medicaid Services
CMMI: Center for Medicare and Medicaid Innovation
COE: Center of Excellence
EHR: Electronic Health Record
FDA: Food and Drug Administration
GARD: Genetic and Rare Diseases Information Center
IAMRARE: “I Am Rare” Registry (Rare Disease Research Platform)
IDD: Intellectual and Developmental Disabilities
NIH: National Institutes of Health
NP: Nurse Practitioner
NPPACT: National Partnership for Pediatric to Adult Care Transition
NORD: National Organization for Rare Disorders
OI: Osteogenesis Imperfecta
OIF: Osteogenesis Imperfecta Foundation
PA: Physician Assistant
PCP: Primary Care Provider
PCORI: PatientCentered Outcomes Research Institute
RCT: Randomized Controlled Trial
RDCADAP: Rare Disease Cures Accelerator – Data and Analytics Platform
US: United States
YAMC: Young Adults with Medical Complexity
