# Supplementary material for: Somewhere to go: a position paper on addressing gaps in transition care for adults with childhood-onset rare diseases
Source: Orphanet J Rare Dis. 2025 Oct 2;20:498. doi: 10.1186/s13023-025-03973-0 (PMC12492944; doi:10.1186/s13023-025-03973-0)
Supplement: Supplementary file 1 — Supplementary Material 1 [file 13023_2025_3973_MOESM1_ESM.docx]

**Appendix**

**Appendix 1: Recommendations Gottransition**

| **Topic** | **Priority** | **Recommendation** |
| --- | --- | --- |
| Prepare the Future Health Workforce to Care for Young Adults with Medical Complexity (YAMC) | 1 | Require medical, nurse practitioner (NP), and physician assistant (PA) students and residents in family medicine and internal medicine to have a minimum exposure to the needs of complex patients with childhood-onset medical conditions through curriculum and experiential learning opportunities. |
|  | 2 | Establish fellowships for adult PCPs, NPs, and PAs focused on YAMC to build a cadre of leaders in care,  research, and policy. |
| Support the Current Adult Primary Care Workforce to Care for YAMC | 3 | Build on existing training infrastructure supported by the federal government to train and provide  technical assistance to existing and future PCPs related to care for YAMC. |
|  | 4 | Create state or regional Centers of Excellence for the care of YAMC to facilitate knowledge sharing among pediatric and adult PCPs and specialists providing care for YAMC. |
| Payment Policy to Support Adult PCPs in Serving YAMC | 5 | Establish payment arrangements that incentivize and support PCPs in providing care to meet the care  delivery needs of YAMC. |
|  | 6 | Embed care coordination and other infrastructure supports within all payment models for adult PCPs  serving YAMC. |
|  | 7 | CMS should use its Innovation (CMMI) Center funding and state Medicaid agencies should use their existing authorities to support payment and delivery models that promote safe, effective, and integrated adult primary care for YAMC. |
| Support for Research to Improve Care of YAMC | 8 | Increase federal research support to build the evidence base for primary care delivery and related  workforce strategies for YAMC, including through the establishment or enhancement of an existing research  center. |
|  | 9 | Establish a Pediatric and Adult Complex Care Research Network to improve continuity of care between  pediatric and adult care and to efficiently expand and assess adult primary care capacity to serve the growing  population of YAMC. |
| Build a Coalition of Interested Organizations to Support Expanded Adult Primary Care Capacity for YAMC | 10 | Convene a series of meetings with key stakeholders interested in improving adult primary care services  available to YAMC to present findings and recommendations from this report and to build a coalition to support implementation and follow up activities. |


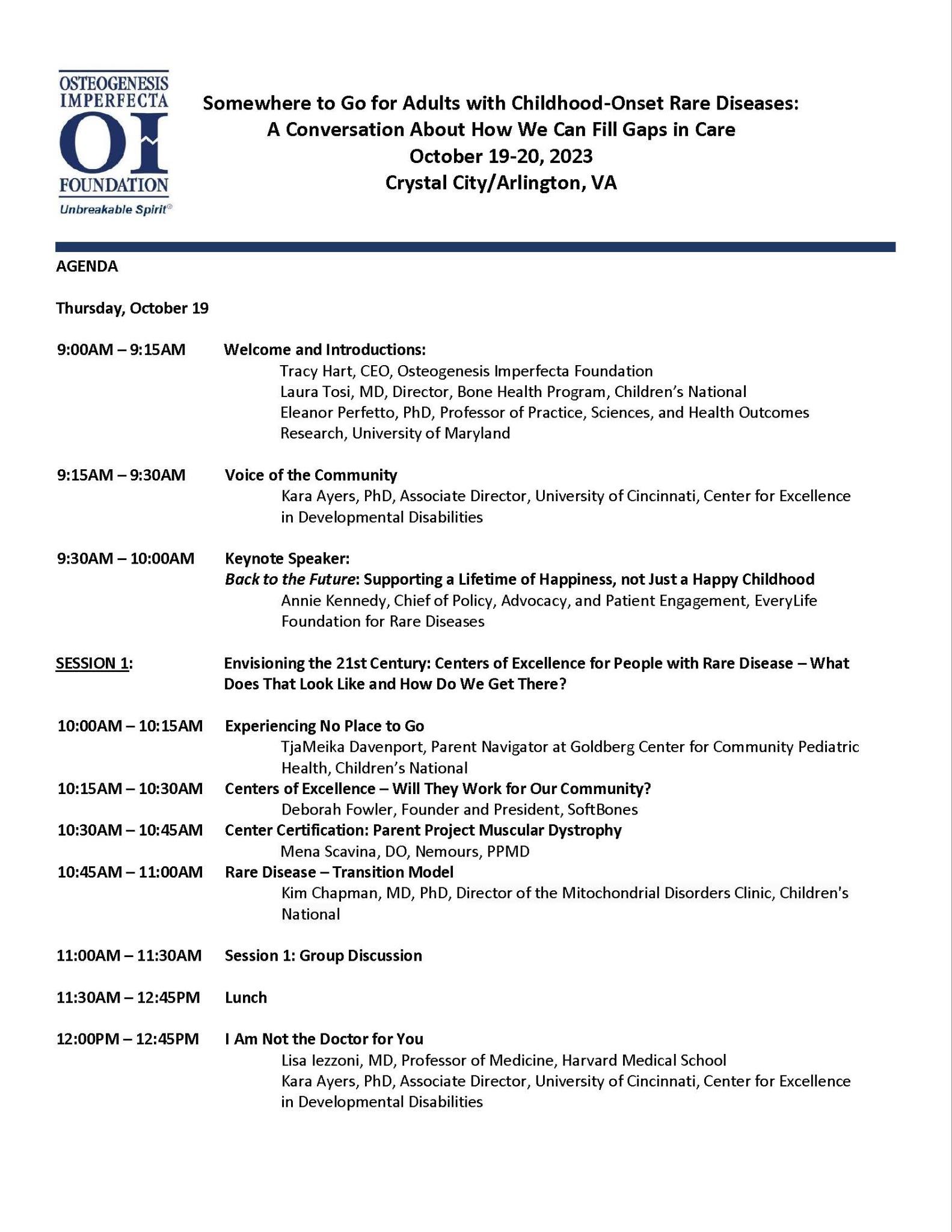

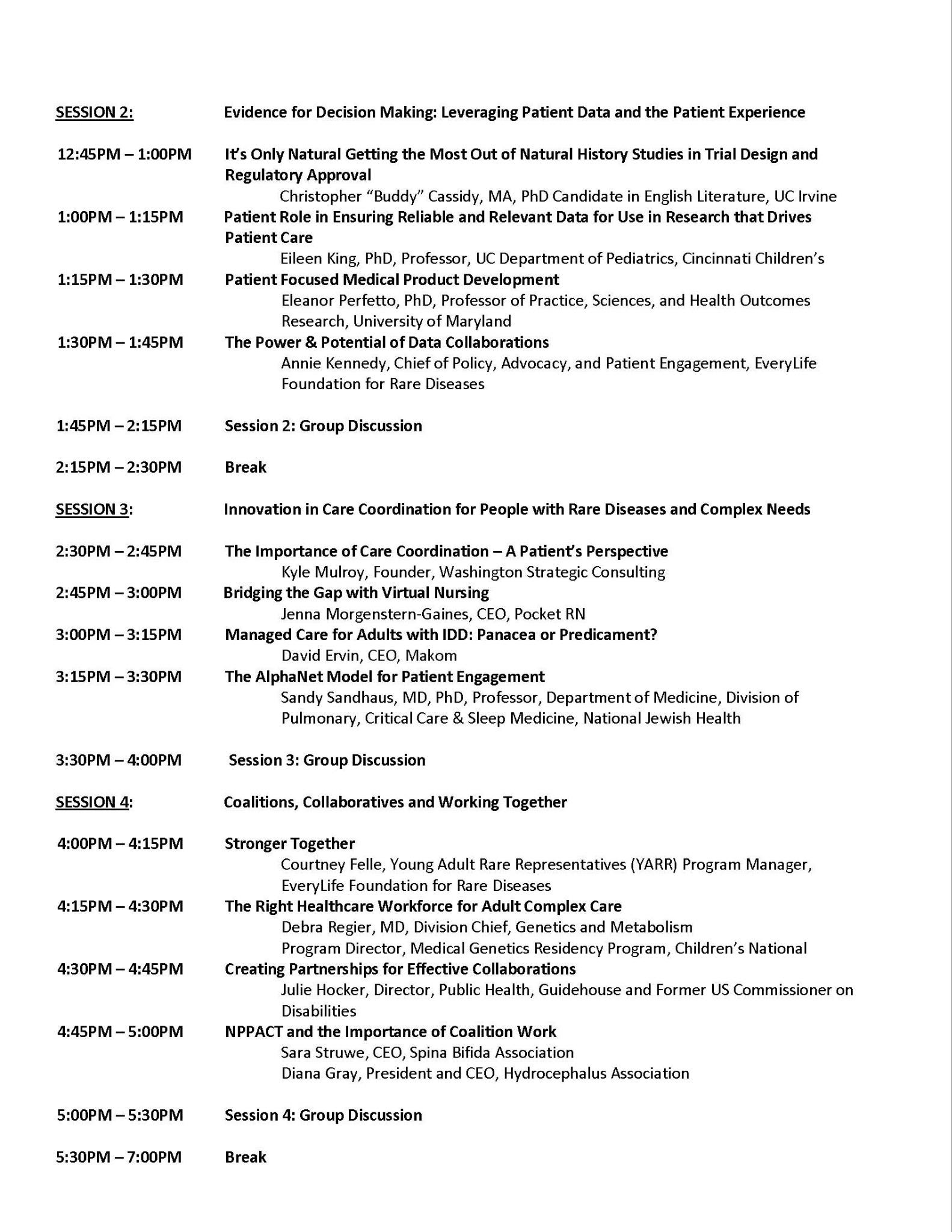

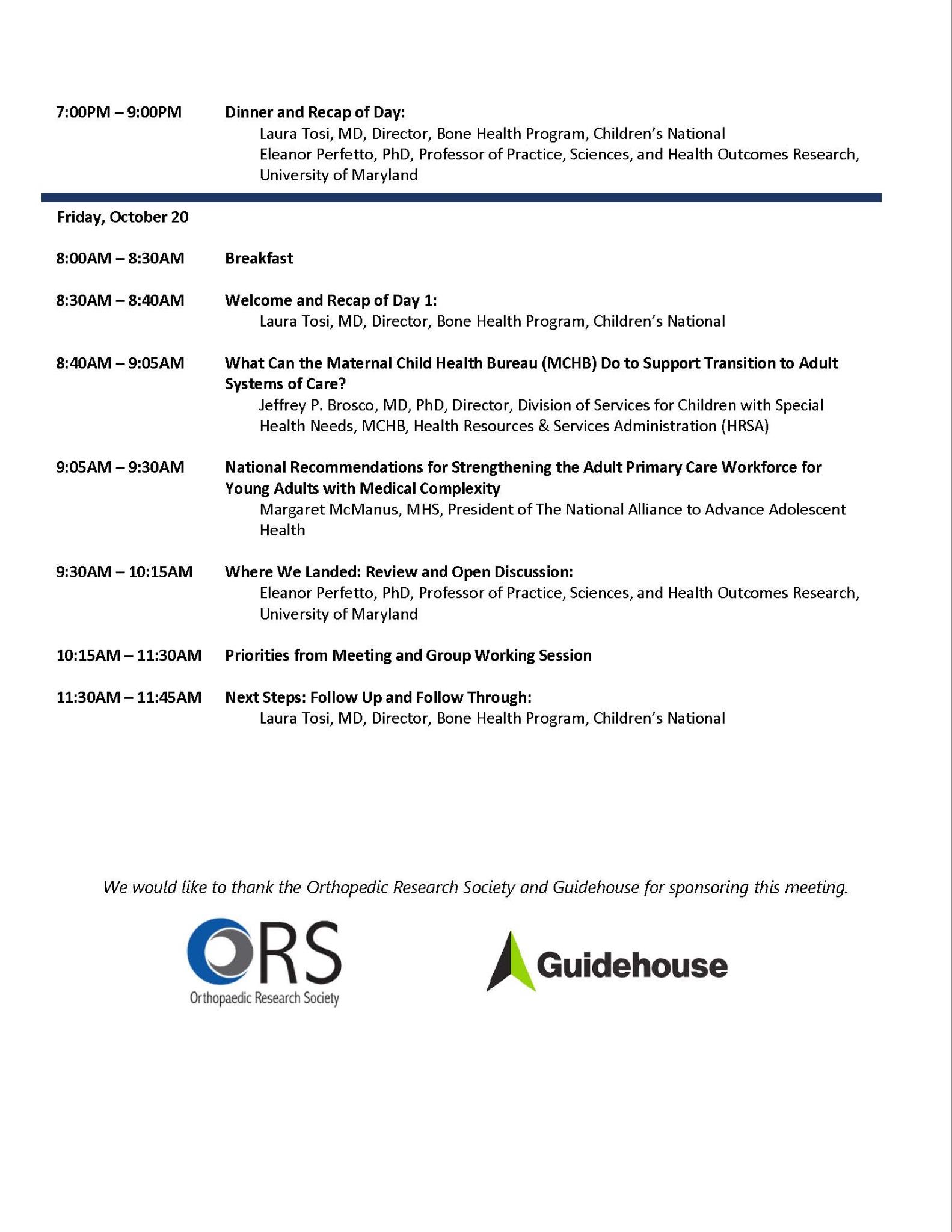


**Appendix 2**

Agenda
